# Guanine-nucleotide exchange on ribosome-bound elongation factor G initiates the translocation of tRNAs

**DOI:** 10.1186/jbiol24

**Published:** 2005-06-27

**Authors:** Andrey V Zavialov, Vasili V Hauryliuk, Måns Ehrenberg

**Affiliations:** 1 Department of Cell and Molecular Biology, Biomedical Center, Uppsala University, SE-75124 Uppsala, Sweden

## Abstract

**Background:**

During the translation of mRNA into polypeptide, elongation factor G (EF-G) catalyzes the translocation of peptidyl-tRNA from the A site to the P site of the ribosome. According to the 'classical' model, EF-G in the GTP-bound form promotes translocation, while hydrolysis of the bound GTP promotes dissociation of the factor from the post-translocation ribosome. According to a more recent model, EF-G operates like a 'motor protein' and drives translocation of the peptidyl-tRNA after GTP hydrolysis. In both the classical and motor protein models, GDP-to-GTP exchange is assumed to occur spontaneously on 'free' EF-G even in the absence of a guanine-nucleotide exchange factor (GEF).

**Results:**

We have made a number of findings that challenge both models. First, free EF-G in the cell is likely to be in the GDP-bound form. Second, the ribosome acts as the GEF for EF-G. Third, after guanine-nucleotide exchange, EF-G in the GTP-bound form moves the tRNA_2_-mRNA complex to an intermediate translocation state in which the mRNA is partially translocated. Fourth, subsequent accommodation of the tRNA_2_-mRNA complex in the post-translocation state requires GTP hydrolysis.

**Conclusion:**

These results, in conjunction with previously published cryo-electron microscopy reconstructions of the ribosome in various functional states, suggest a novel mechanism for translocation of tRNAs on the ribosome by EF-G. Our observations suggest that the ribosome is a universal guanosine-nucleotide exchange factor for EF-G as previously shown for the class-II peptide-release factor 3.

## Background

**Figure 1:**
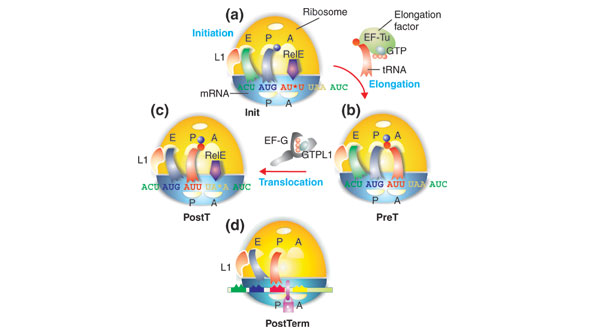
Schematic representation of **(a) **initiation, **(b) **pre-translocation, **(c) **post-translocation, and **(d) **post-termination complexes, referred to as Init, preT and postT, and postTerm, respectively. A, amino-acyl tRNA site on the ribosome; P, peptidyl-tRNA site; E, exit site; L1, ribosomal protein. The large subunit of the ribosome is shown in yellow and the small subunit in blue. The colored ribbons represent tRNAs and the colored balls represent amino acids in aminoacyl- or peptidyl-tRNA. The purple arrow represents RelE, which cleaves the codon shown at the *. The mauve padlock in (d) illustrates a state of the ribosome in which the mRNA is locked, and cannot move in relation to the small subunit. The figure represents a special case in which the postT ribosome has a stop codon (UAA) in the A site, and is therefore also a pre-termination (preTerm) ribosome. For further details see text and Figure 8.

During the translation of protein, in every peptide elongation cycle, one aminoacyl-tRNA arrives at and binds to the A site of the ribosome. Then, peptidyl transfer brings the ribosome to its pre-translocation (preT) state, with a peptidyl-tRNA in the A site (Figure 1a,b). Subsequent translocation of the complex comprising two charged tRNAs and the mRNA – the tRNA_2_-mRNA complex – to the post-translocation state (postT) (Figure 1c) completes the elongation cycle. In bacteria, translocation of peptidyl-tRNA from the A site to the P site of the ribosome is catalyzed by elongation factor EF-G (Figure 1b,c). Like the ribosomal GTPases RF3, EF-Tu and IF2, EF-G belongs to the family of small GTPases [1]. Conserved features of the GTP-binding domain of these protein factors are responsible for their function as molecular switches [2]. In the active GTP-bound conformation, the GTPases bind tightly to their targets. After GTP hydrolysis, they adopt an inactive GDP-bound conformation, and dissociate rapidly from their targets [1]. Such GTPases usually require a guanine-nucleotide exchange factor (GEF), which catalyzes the exchange of GDP to GTP, and a GTPase-activating protein (GAP), which stimulates GTP hydrolysis [2]. In the case of EF-G, the role of GAP has been ascribed to the ribosomal L7/L12 stalk [3]. No GEF has so far been identified for EF-G, however, and it has been postulated that rapid and extensive exchange of GDP to GTP occurs spontaneously on free EF-G [3]. Accordingly, it has been assumed that EF-G is in the GTP-bound form as it enters the ribosome, although this structure has eluded detection in solution [4], and has only been observed in ribosomal complexes [5].

According to the 'classical' model, the binding of EF-G•GTP to the preT ribosome complex (Figure 1b) promotes translocation of the peptidyl-tRNA from the A to the P site. Then, GTP hydrolysis removes the EF-G from the postT ribosome [4,6]. Recent experiments, suggesting that GTP hydrolysis on EF-G precedes translocation and that EF-G together with GDP can promote rapid translocation, have led to the contrasting suggestion that EF-G is in fact a 'motor protein' that drives translocation with the energy liberated by GTP hydrolysis [7]. Previously, we showed that the postT ribosome complex has low affinity for EF-G•GTP [8], presumably as a result of the inability of a peptidyl-tRNA to be accommodated in a hybrid P/E tRNA site, where the CCA-end of the tRNA is in the E site of the large ribosomal subunit, and the anticodon-end of the tRNA is in the P site of the small ribosomal subunit. This effectively prevents formation of the 'twisted' ribosome conformation [5] with a high affinity for the GTP form of EF-G. These results also show that translocation cannot be carried out by EF-G and GDP, in line with the notion that EF-G, like other small GTPases, has an active GTP- and inactive GDP-bound form [1].

In this study, we challenge current ideas about the mechanism of translocation. The paradigm shift we propose follows from our observations that intracellular EF-G is likely to be in the GDP-bound form, that the GDP form of the factor can rapidly enter the preT ribosome complex, and that the preT ribosome acts as the GEF for EF-G, similar to the way that the post-termination ribosome acts as the GEF for the peptide-release factor RF3 [9,10]. Our results, partially based on the use of A-site-specific cleavage of mRNA by the bacterial toxin RelE [11] to monitor the position of the mRNA at various translocation steps, show that the exchange of GDP for the non-cleavable GTP analog GDPNP on EF-G bound to the preT complex drives the ribosome into an intermediate translocation state (transT*), wherein the tRNA_2_-mRNA complex has moved in relation to the 30S subunit. The removal of EF-G•GDPNP from a transT* ribosome by addition of excess GDP brings the ribosome back to its preT state, while GTP addition brings it to the postT state. From these and previous biochemical data [8], in conjunction with cryo-electron microscopy (cryo-EM) reconstructions of functional ribosomal complexes [5], we provide a mechanistic reinterpretation of the major steps of translocation.

## Results

### The ribosome is the missing guanine-nucleotide exchange factor for EF-G

It has been reported that EF-G from *Escherichia coli *binds to GTP with ten-fold lower affinity than it binds to GDP [12]. On the assumption that there is a ten-fold excess of GTP over GDP in the cytoplasm and rapid nucleotide exchange on free EF-G, it was suggested that the rate-limiting step of guanine-nucleotide exchange in the EF-G cycle is the dissociation of EF-G•GDP from the postT ribosome [3].

**Figure 2:**
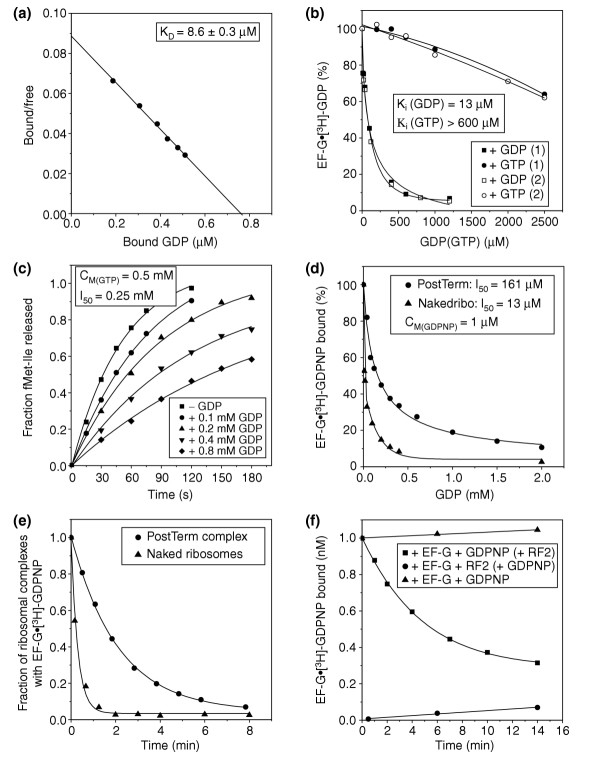
Ribosome-dependent exchange of GDP to GTP on EF-G. **(a) **Scatchard plot from a nitrocellulose-filtration experiment to obtain the dissociation constant for the binding of [^3^H]-GDP to free EF-G. **(b) **Chase of [^3^H]-GDP from free EF-G by unlabeled GTP or, as a control, GDP. The dissociation constant for GTP binding to free EF-G was obtained from the corresponding constant for GDP binding in (a) and from the inhibition of [^3^H]-GDP binding to EF-G by GTP addition. The figure shows the results of two independent experiments (1 and 2). **(c) **Time-dependent release of fMet-Ile by 0.5 μM RF2 after translocation of fMet-Ile-tRNA^Ile ^from the A to the P site by a catalytic amount of EF-G (10 nM) added to 23 nM preT ribosomes together with 0.5 mM GTP and 0–0.8 mM GDP. CM(GTP) is the GTP concentration and I_50_ is the GDP concentration at which the rate of translocation is reduced to half-maximal value. **(d) **Inhibition of EF-G•GDPNP binding to post-termination (PostTerm) complexes or naked 70S ribosomes (Nakedribo) in the presence of 1 μM [^3^H]-GDPNP and 0–2 mM unlabeled GDP. **(e) **Fraction of [^3^H]-GDPNP (total concentration 1 μM) bound to EF-G• [^3^H]-GDPNP in postTerm complexes or in naked ribosomes as a function of time after addition of unlabeled GDP to a concentration of 2 mM. **(f) **Time-dependence of EF-G• [^3^H]-GDPNP binding to postTerm ribosomes in the presence of 1 μM [^3^H]-GDPNP: in the absence of RF2 (control), after addition of [^3^H]-GDPNP to EF-G pre-incubated with RF2 and postTerm ribosomes, or after addition of RF2 to EF-G pre-incubated with [^3^H]-GDPNP and postTerm ribosomes.

Our earlier data, showing the ribosome to be a GEF for RF3 [9], prompted us to re-check the binding of free EF-G to GDP or GTP. The dissociation constant (K_D_) for the EF-G•GDP complex was about 9 μM (Figure 2a), close to an earlier estimate of 4 μM [12]. Results from experiments in which [^3^H]-GDP in complex with EF-G was chased with unlabeled and further purified GTP [9] (see below and Figure 4a for purification details), however, show a 60-fold larger effective K_D_-value for the binding of EF-G to GTP than to GDP (Figure 2b). This factor of 60 provides a lower boundary to the correct value, because purified GTP solutions do contain some fraction of GDP from the hydrolysis of GTP. The intracellular GTP:GDP ratio has been estimated as 7:1 for *Salmonella enterica *serovar Typhimurium [13], and is probably similar in *E. coli. *This suggests that a major fraction of free EF-G in *E. coli *is bound to GDP.

If binding of EF-G to the pre-translocation (preT) ribosome required the factor to be bound to GTP, this would significantly reduce the rate of association of EF-G with the ribosome. This problem would, however, be eliminated if EF-G in the GDP-bound form associated rapidly with the ribosome and GDP-to-GTP exchange took place on, rather than off, the ribosome. To test the latter two hypotheses, we prepared preT ribosomes with fMet-Ile-tRNA^Ile ^and its corresponding codon in the A site and a UAA stop codon immediately downstream from the Ile codon. Translocation was catalyzed by EF-G at such a small concentration that each EF-G molecule had to cycle many times to obtain a significant fraction of translocated ribosomes. The concentration of GTP was fixed at 0.5 mM during incubations with varying concentrations of GDP, and the ribosome concentration chosen was sufficiently low that the rate of translocation per ribosome was approximated by the concentration of free EF-G multiplied by its effective association rate constant (*k*_*cat*_/*K*_*m*_) for ribosome binding (see Materials and methods). Because translocation brought the stop codon UAA into the ribosomal A site, the extent of translocation was conveniently quantified as the fraction of fMet-Ile peptide that could be rapidly released by RF2, when RF2 was added to a concentration in excess of that of the ribosomes at varying incubation times (Figure 2c).

We obtained 50% inhibition of the rate of EF-G recycling at 0.25 mM GDP, at which concentration the concentration of EF-G•GDP (K_D _= 9 μM) must have been at least 30 times larger than the concentration of EF-G•GTP (K_D _> 0.6 mM). If entry of EF-G to the ribosome had required the EF-G•GTP complex, this would have led to a 30-fold, rather than the observed two-fold, inhibition of translocation at 0.25 mM GDP (see Materials and methods). This implies that EF-G must have entered the ribosome in complex with GDP, and that the exchange of GDP for GTP must have taken place on, rather than off, the ribosome. The parameters that determine how the *k*_*cat*_/*K*_*m *_value for the entry of EF-G to the preT ribosome complex depends on varying ratios of GDP to GTP are defined in Materials and methods for a particular kinetic scheme.

The preT ribosome contains a deacylated tRNA in the P site (Figure 1b), which may be important for the GDP-to-GTP exchange reaction. This is suggested by experiments on guanine-nucleotide binding to EF-G in another type of ribosome complex. Here, EF-G was incubated with [^3^H]-GDPNP and either post-termination (postTerm) or naked ribosomes at varying concentrations of unlabeled GDP (Figure 2d). The postTerm ribosome has a deacylated tRNA in the P site and an empty A site programmed with a stop codon (Figure 1d), while the naked ribosome lacks ligands. The fraction of [^3^H]-GDPNP retained on a nitrocellulose filter, corresponding to ribosome-bound EF-G• [^3^H]-GDPNP, was reduced to 50% at a 160-fold excess of GDP in the postTerm case, or a 13-fold excess for the naked ribosomes. This implies that EF-G, bound to either type of ribosome, had much higher affinity for GDPNP than for GDP, and that the difference was more pronounced for postTerm than for naked ribosomes (Figure 2d,e). Accordingly, the presence of a deacylated tRNA in the P site of the preT ribosome led to more stable binding of EF-G• [^3^H]-GDPNP to this complex than to the naked ribosome. A corresponding stabilization of the EF-G•GTP complex on preT ribosomes by the P-site tRNA is expected, and would contribute to efficient guanine-nucleotide exchange (Figure 2c).

So far, we have not addressed the question of whether formation of a complex between EF-G•GDP and the preT ribosome leads directly to guanosine exchange, or whether the exchange reaction is preceded by a change in conformation of the ribosome. This problem is addressed in the next section.

### EF-G•GDP drives the preT ribosome into a state that has hybrid tRNA sites

EF-G•GTP binds poorly to the pre-termination (preTerm) ribosome with a peptidyl-tRNA in the P site and an empty A site programmed with a stop codon (Figure 1c), but binds with high affinity to the postTerm ribosome with a deacylated tRNA in the P site [8] (Figure 1d). In the latter case, cryo-EM results show the postTerm ribosome in a ratcheted state with the P-site tRNA in the hybrid P/E site [5]. This suggests that high-affinity binding of EF-G•GTP to the ribosome requires the ratcheted state with hybrid tRNA sites; this state cannot be formed when there is peptidyl-tRNA in the P site. It is likely that the ratcheted ribosome conformation appears also in the translocation process, suggesting that EF-G•GDP can move the preT ribosome from the relaxed state, with three full binding sites for the tRNAs [5], to the ratcheted state, with no E site binding and only two binding sites for tRNA [14]. This would facilitate rapid GDP-to-GTP exchange on EF-G, and we have tested one of the predictions that emerges from this hypothesis, namely that the apparent affinity of a deacylated tRNA for the E site of the preT ribosome will be reduced by the addition of EF-G•GDP. This prediction was confirmed by an experiment showing that the affinity of tRNA^fMet ^for the E site of the preT ribosome was successively reduced by increasing amounts of EF-G in the presence of GDP (Table 1, set 1).

In order to monitor the translocation events that follow guanine-exchange on EF-G on the preT ribosome, we used A-site-specific cleavage of the mRNA by the bacterial toxin RelE, and this is described next.

### Translocation events monitored by RelE cleavage of the A-site codon

**Figure 3:**
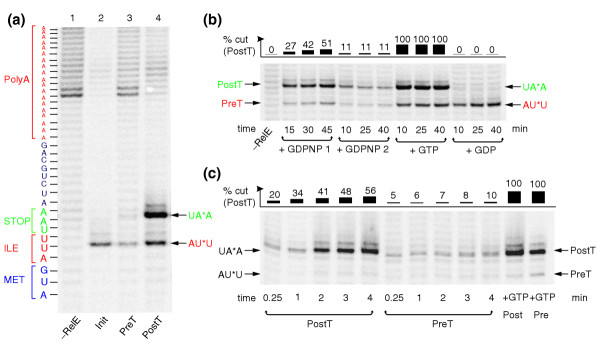
RelE cleavage of mRNA in the A site of ribosomal complexes. **(a) **The mRNA fragments resulting from RelE cleavage in the A site of the three ribosomal complexes Init (see Figure 1a), preT (see Figure 1b) and postT (see Figure 1c), separated on a 10% sequencing gel. The amount of radioactivity in the postT lane was doubled to make the AUU cleavage visible. **(b) **Time-dependent cleavage of mRNA by RelE; preT ribosomes were incubated with EF-G together with GDPNP (+ GDPNP 2) or GTP (+ GTP) or GDP (+ GDP). RelE was added after 10, 25 or 40 min, and the reaction was in each case quenched 5 min after RelE addition. Alternatively, preT ribosomes were incubated together with EF-G, RelE and GDPNP and the reaction quenched after 15, 30 or 45 min (+ GDPNP 1). **(c) **Time-dependent cleavage of mRNA by 120 nM RelE in the A site of 0.3 μM postT or preT ribosome complexes incubated with 2 μM EF-G and 0.6 mM GDPNP. As a control, in the last two lanes 1 mM GTP was added to postT or preT ribosomes at the end of the incubation.

RelE cuts mRNA specifically within the ribosomal A site [11], and we used this activity to monitor ribosome movement along mRNA in the translocation steps (Figure 1). An initiation complex (Init; Figure 1a) with fMet-tRNA^fMet ^in the P site was constituted by incubating ribosomes in the presence of initiation factors IF1, IF2 and IF3, fMet-tRNA^fMet^, and ^33^P-end-labeled mRNA encoding the dipeptide Met-Ile-stop (AUG AUU UAA). Exposure of this complex to RelE led to unique cleavage of the A-site codon to AU*U (Figure 3a, lane 2). The Init complex (Figure 1a) was then converted to the preT complex (Figure 1b) by addition of the ternary EF-Tu•GTP•Ile-tRNA^Ile ^complex. The resulting presence of fMet-Ile-tRNA^Ile ^in the A site blocked the entry of RelE to the A site and reduced the rate of cleavage of the AUU codon (Figure 3a, lane 3). Addition of EF-G•GTP to the preT complex catalyzed rapid translocation of fMet-Ile-tRNA^Ile ^from the A to the P site, generating the postT complex (Figure 1c), and moved the stop codon into the A site of the postT complex, where it was rapidly cleaved by RelE (Figure 3a, line 4).

### Complete translocation requires GTP and GTP hydrolysis

In order to study further the guanine-nucleotide dependence of the translocation steps, the ribosomal preT complex was first separated from all other components of the translation mixture [8]. RelE cleavage of the A-site codon was monitored after addition of EF-G to the purified preT complex in the presence of GTP, GDP or the non-cleavable GTP analog GDPNP (Figure 3b). In one type of experiment, the preT complex was first incubated with EF-G and either GTP or GDP for 10, 25 or 40 min and then the ribosomes were exposed to RelE for 5 min. In the presence of GTP, there was extensive cleavage by RelE of the stop codon (Figure 3b, + GTP), meaning that a major fraction of the ribosomes had moved from the preT to the postT state.

**Figure 4:**
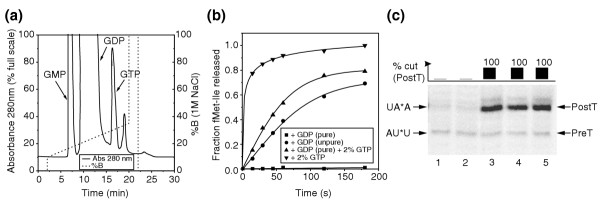
Contamination of GDP preparations with GTP strongly stimulates translocation by EF-G. **(a) **Elution profile of commercially available GDP from a MonoQ column showing the GTP and GMP contaminations. %B is the percentage of buffer B (20 mM Tris-HCl, 1 M NaCl) in the buffer A (20 mM Tris-HCl) + B mixture. **(b) **Time-dependent release of peptide by 0.4 μM RF2 after translocation of fMet-Ile-tRNA (23 nM total) from the A site to the P site by 1 μM EF-G in the presence of 1 mM purified GDP, unpurified GDP, purified GDP containing 20 μM GTP (2%), or 20 μM GTP. **(c) **Cleavage of mRNA by RelE incubated with 0.15 μM preT, 2 μM EF-G and nucleotides. Lanes: (1) no GDP; (2) 1 mM purified GDP; (3) 1 mM unpurified GDP; (4) 1 mM purified GDP containing 2% GTP; (5) 20 μM GTP.

In the presence of GDP, there was no significant RelE-dependent cleavage of the stop codon in the A site, even during the longest incubation time of 45 minutes (Figure 3b, + GDP), meaning that the ribosomes had remained in their preT state during the whole incubation period. This implies that EF-G and GDP were unable to promote translocation, in apparent contradiction to previous results, showing rapid translocation by EF-G and GDP [7]. We have noted that GTP contamination, common in commercial preparations of GDP, can have profound effects on the GTPases of protein synthesis. A typical elution profile (Figure 4a) shows such a GDP preparation to contain between 1 and 2% GTP, and the effect of this low level of contamination was studied in an experiment in which translocation of fMet-[^14^C]-Ile-tRNA from the A site to the P site was probed by the fraction of peptide that could be rapidly released by RF2. The rate of translocation was insignificant with purified GDP, intermediate with unpurified GDP or with purified GDP + 2% GTP and fast with GTP (Figure 4b). Similarly, no translocation with pure GDP was detected by assessing the RelE-dependent cleavage of the mRNA (Figure 4c). Our nucleotide preparations were further purified by ion exchange chromatography on a MonoQ column [9], while those of Rodnina *et al. *[7] were not. This suggests that their 'GDP-dependent translocation' was, in fact, due to contaminating GTP. At such a large excess of GDP, the guanine-exchange reaction on the preT ribosome is expected to be the rate-limiting step for translocation, and this will lead to slow, monophasic translocation, exactly as they observed (see Materials and methods) [7].

In the presence of GDPNP, about 11% of the stop codons were cleaved after addition of RelE, irrespective of the time of exposure of preT ribosomes to EF-G and GDPNP (Figure 3b, + GDPNP 2). In a similar experiment, modified so that RelE was present from the start of the incubation of preT ribosomes with EF-G and GDPNP, the fraction of cleaved stop codons increased slowly with time (Figure 3b, + GDPNP 1). This means that EF-G and GDPNP drove the ribosomes to a state that remained stable during the 45 min incubation in the absence of RelE (Figure 3b, + GDPNP 1). In this state, the stop codon was partially available for RelE-mediated cleavage in the A site, resulting in very slow truncation of the mRNA (Figure 3b, + GDPNP 2). *A priori*, this ribosomal state could be the postT state of the ribosome or a novel transition state ('transT*') in the translocation process where, in both cases, RelE-mediated cleavage of the stop codon in the A site was inhibited by ribosome-bound EF-G•GDPNP. An experiment in which the rates of RelE cleavage in the A-site codons of ribosomes in the putatively new state and postT ribosomes were compared at the same concentrations of EF-G and GDPNP (Figure 3c) showed that RelE cleaved the mRNA in the postT complex much faster than the mRNA on the ribosomes in the unknown state complex, proving that the ribosomal complexes could not have been the same. This means that the unknown state was transT*, and in the next section we characterize these complexes with respect to tRNA-exchangeability.

### Exchangeability of tRNA^fMet ^in preT, transT* and postT ribosomes

**Figure 5:**
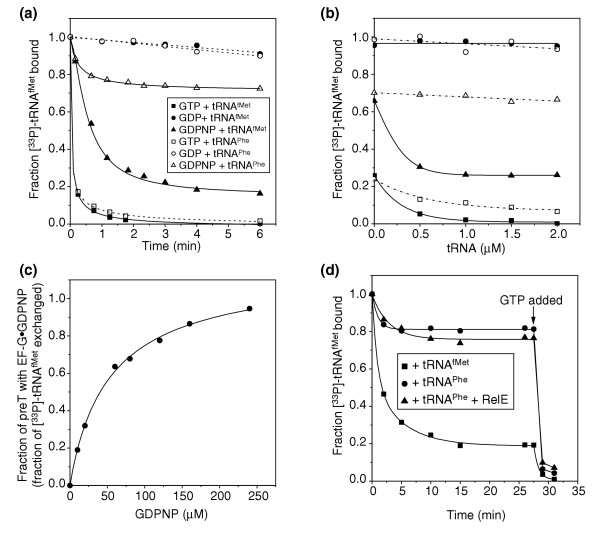
Properties of the transition state. **(a) **Time-dependent exchange of [^33^P]-tRNA^fMet ^bound to the P site of 70 nM preT complex with 1 μM unlabeled tRNA^fMet ^or tRNA^Phe ^after the addition of 2 μM EF-G and 1 mM nucleotide. **(b) **The fraction of [^33^P]-tRNA^fMet ^exchanged with tRNA^fMet ^or tRNA^Phe ^after 9 min incubation of 70 nM preT with 2 μM EF-G, 1 mM nucleotide and 0–2 μM tRNA^fMet ^or tRNA^Phe^. **(c) **Fraction of [^33^P]-tRNA^fMet ^on 88 nM preT ribosomes exchanged after 7 min incubation with 2 μM unlabeled tRNA^fMet^, 2 μM EF-G and 0–240 μM GDPNP to estimate the fraction of ribosomes containing EF-G•GDPNP. **(d) **Exchange of [^33^P]-tRNA^fMet ^with 2 μM tRNA^fMet ^or tRNA^Phe ^added to 78 nM preT incubated with 2 μM EF-G, 0.4 nM GDPNP with or without 80 nM RelE. At 27.5 min, 1 mM GTP was added to translocate [^33^P]-tRNA^ fMet ^to the E site.

We characterized the transT* state with respect to the exchangeability of its deacylated tRNA^fMet^. First, we used nitrocellulose filtration to study dissociation of [^33^P]-tRNA^fMet^, originally in the P site of the preT complex (Figure 1b), from ribosomes incubated with EF-G together with GDP, GTP or GDPNP. In one type of experiment, the fraction of ribosome-bound [^33^P]-tRNA^fMet ^was monitored as a function of time in the presence of either unlabeled tRNA^fMet ^or tRNA^Phe ^at fixed concentrations (Figure 5a). In another type of experiment, the fraction of ribosome-bound [^33^P]-tRNA^fMet ^was monitored at a fixed time while varying the concentrations of unlabeled tRNA^fMet ^or tRNA^Phe ^(Figure 5b).

In the GDP experiment in which no translocation occurred (Figure 3b, + GDP), there was no significant removal of [^33^P]-tRNA^fMet ^from the ribosome during 6 min in the presence of any unlabeled tRNA, as would be expected for ribosomes with deacylated tRNA^fMet ^stably bound to the P site after peptidyl transfer (Figure 5a,b). In the GTP case, in which there was rapid translocation (Figure 3b, + GTP), there was fast dissociation of [^33^P]-tRNA^fMet ^in the presence of either tRNA^fMet ^or tRNA^Phe ^(Figure 5a). The titration experiment (Figure 5b) shows that one fraction of [^33^P]-tRNA^fMet ^dissociated from the postT ribosomes in the absence of chasing tRNAs, and that the remaining fraction could be titrated out with either tRNA^fMet ^or tRNA^Phe^. These results reflect the comparatively low affinity of [^33^P]-tRNA^fMet^for the E site and the lack of codon specificity for the E-site-bound tRNAs ([15]; see also below).

In the case of GDPNP, [^33^P]-tRNA^fMet ^dissociated slowly in the presence of tRNA^fMet^, but there was no dissociation in the presence of tRNA^Phe^, suggesting high affinity for [^33^P]-tRNA^fMet ^and retained codon-specificity for deacylated tRNAs (Figure 5a). In line with this, the titration experiment (Figure 5b) shows that [^33^P]-tRNA^fMet ^could be exchanged with unlabeled tRNA^fMet ^but not with unlabeled tRNA^Phe^.

In a third type of experiment, [^33^P]-tRNA^fMet ^was chased with unlabeled tRNA^fMet ^from preT ribosomes incubated for a fixed amount of time in the presence of EF-G at a constant concentration and GDPNP at varying concentrations (Figure 5c). The fraction of ribosomes lacking [^33^P]-tRNA^fMet ^increased from 0 to 50% when GDPNP was varied from 0 to 40 μM and increased further to almost 100% at 250 μM GDP. This result shows that the affinity of EF-G•GDPNP for the transT* ribosome, containing one deacylated and one peptidyl tRNA, was approximately 100 times weaker than the affinity of EF-G•GDPNP for the postTerm ribosome, containing only one deacylated tRNA (see below)[8].

Another experiment (Figure 5d) shows that tRNA^Phe ^could not replace [^33^P]-tRNA^fMet ^in transT* ribosomes, either with intact mRNA or with mRNA that had been cleaved by RelE. This means that the transT* ribosomes did not move to the postT state as a result of the mRNA cleavage, since that would have resulted in weak, non-selective E-site binding of the deacylated tRNAs (as shown in Table 1 and Figure 7).

### Addition of GDP to transT* ribosomes brings them back to the preT state

**Figure 6:**
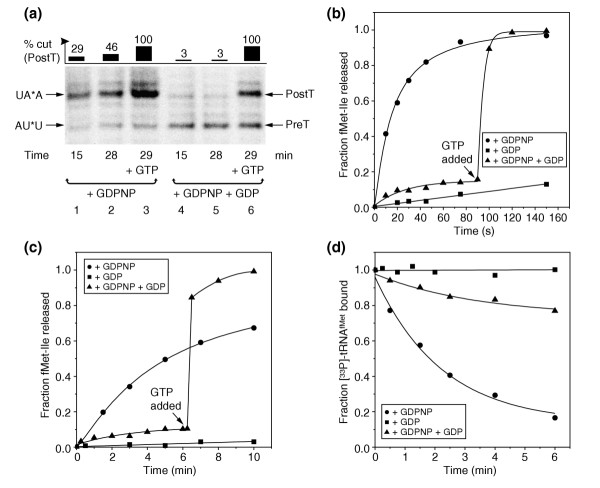
Removal of EF-G•GDPNP from the transition state with GDP. **(a) **Time-dependent cleavage of mRNA by 166 nM RelE in transT* complex in the presence of 2 μM EF-G and 0.32 mM GDPNP (GDPNP case) or after further addition of GDP to a concentration of 1 mM to remove EF-G from the ribosome (GDPNP + GDP case). In each case, GTP was added to a final concentration of 1 mM at 29 min to show the fraction of ribosomes that was active in translocation (lanes 3 and 6). **(b,c) **Time-dependent release of fMet-Ile by (b) 0.4 mM puromycin or (c) 0.5 μM RF2; 2 μM EF-G was pre-incubated with 46 nM preT complex and 40 μM GDPNP or polymix buffer for 3 min at 37°C. Then, buffer or 2 mM GDP was added and the incubation was continued for 1 min. Finally, (b) 0.4 mM puromycin or (c) 0.5 μM RF2 was added and the extent of peptide release was observed over time. **(d) **Exchange of [^33^P]-tRNA^fMet ^on 88 nM preT complex, pre-incubated with 2 μM EF-G and 100 μM GDPNP or with buffer, with 2 μM tRNA^fMet ^in the presence or absence of 2 mM GDP.

When GDP was added to transT* ribosomes, on which we have observed RelE-mediated cleavage of the stop codon to UA*A (Figure 3b, + GDPNP 1; and Figure 6a, + GDPNP), stop codon cleavage was completely eliminated and replaced by cleavage of the AUU codon (Figure 6a, + GDPNP + GDP). The latter cleavage reaction was typical for the preT ribosome and occurred when the peptidyl-tRNA dissociated from the A site (Figure 3a). When, in contrast, GDP was added to postT ribosomes that were incubated in the presence of EF-G and GDPNP, the ribosomes remained in the postT state and there was rapid cleavage of the UAA codon (data not shown). These results strongly suggest that addition of GDP to the transT* ribosome brought it back to the preT state, providing further evidence that the transT* state is different from the postT state of the ribosome.

In line with previous results [8], addition of EF-G•GDPNP to preT ribosomes brought them to a puromycin-reactive state (Figure 6b); puromycin mimics an aminoacyl tRNA and removes a nascent peptide from the ribosome by acting as a receptor in peptidyl-transfer. When GDP was also included, however, the puromycin-reactivity of the ribosomes was lost (Figure 6b), again showing that the resulting state could not have been the postT ribosome, which is fully reactive to puromycin [9].

A deacylated [^33^P]-tRNA^fMet ^in the transT* ribosome could readily be chased with unlabeled tRNA^fMet^, but its exchange rate in the preT ribosome was almost zero (Figure 5a,b). If GDP addition brought the transT* ribosome back to the preT state, one would therefore expect the exchange rate of the tRNA^fMet ^to drop drastically. This prediction was nicely confirmed by experiments showing that addition of GDP to transT* ribosomes did indeed prevent exchange of [^33^P]-tRNA^fMet ^with tRNA^fMet ^(Figure 6d).

When release factor RF2 was added to transT* ribosomes, there was slow release of peptide (Figure 6c), suggesting that there was partial availability of the UAA stop codon in the A site, a necessary condition for termination by class-1 release factors [8]. Addition of GDP to transT* ribosomes made them non-reactive not only to puromycin (Figure 6b), but also to peptide release induction by RF2 (Figure 6c). These mRNA cleavage results (Figure 6a), along with those for puromycin (Figure 6b), RF2 (Figure 6c) and tRNA exchange (Figure 6d) show that removal of EF-G•GDPNP from the transT* ribosome by the addition of GDP brought the ribosome back to the preT state with peptidyl-tRNA in the A site. This confirms that the transT* state cannot be identical to the postT state of the ribosome, and corroborates that transT* is a transition state in the translocation process, in which rapid hydrolysis of native GTP on EF-G normally occurs. When EF-G dissociated from the transT* ribosome, the mRNA rapidly slipped back to its preT position, but there was a short time during which RelE could cleave and RF1 could interact with the stop codon exposed in an EF-G-free A site.

### Deacylated tRNAs bind to the ribosomal E site with low codon specificity

**Figure 7:**
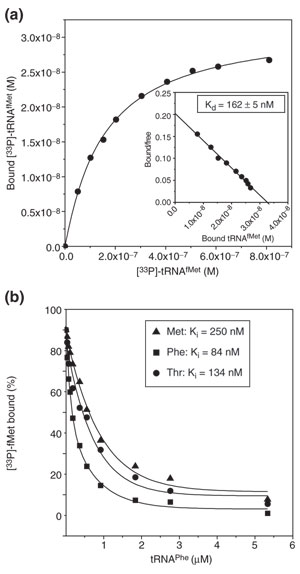
Binding of deacylated tRNA to the E site. **(a) **Binding of [^33^P]-tRNA^fMet ^to the postT complex with fMet-Phe-Ile-tRNA^Ile^in the P site and a Thr codon (ACG) in the E site. Insert: Scatchard plot to obtain the dissociation constant. **(b) **Chase of [^33^P]-tRNA^fMet ^from the E site of postT complexes containing Met (AUG), Phe (UUU) or Thr (ACG) codons with unlabeled tRNA^Phe^. Dissociation constants for [^33^P] tRNA^fMet ^were obtained as in (a) and the dissociation constants for tRNA^Phe ^were calculated from the 50% chase (I_50_) concentrations of tRNA^Phe^.

We showed above that [^33^P]-tRNA^fMet ^could be chased by tRNA^fMet ^but not by tRNA^Phe ^in transT* (Figure 5d). This contrasts with E-site binding of deacylated tRNA, as follows. We designed experiments to obtain dissociation constants for the binding of deacylated tRNA^fMet ^or tRNA^Phe ^to the E site of postT ribosomes, programmed with Met (AUG), Phe (UUU) or Thr (ACG) codons. The binding of [^33^P]-tRNA^fMet ^to the E site was assayed by nitrocellulose filtration, and a representative experiment with the Thr (ACG) codon in the E site is shown in Figure 7a. Dissociation constants for the binding of tRNA^Phe ^or tRNA^Thr ^to the differently programmed E sites of postT ribosomes were obtained as I_50 _values in competition experiments with a constant and almost saturating concentration of [^33^P]-tRNA^fMet ^and varying concentrations of unlabeled tRNA^Phe ^or tRNA^Thr ^(Figure 7b). The outcome of typical experiments, probing the binding of tRNA^Phe ^to E sites programmed with Met, Phe or Thr codons, is shown in Figure 7b, and all data are collected in Table 1. The results show that tRNA^fMet ^and tRNA^Phe ^bound to postT ribosomes with similar affinities and weak codon specificity. In similar experiments, we also found that the affinity of tRNA^fMet ^for the E site was similar for postT ribosomes, postT ribosomes with RelE-mediated cleavage of the mRNA in the A site, and preT ribosomes, all with the same codon in the E site (Table 1).

## Discussion

In this work, we present experimental results that redefine the roles of guanine-nucleotide exchange and GTP hydrolysis on the elongation factor EF-G during translocation of tRNAs on the ribosome. Taking advantage of an *in vitro *system with pure components and high activity, we characterized the different states of the ribosome during translocation of tRNAs and mRNA as catalyzed by EF-G. On the basis of these experimental results we propose a novel mechanism for translocation, a process that is conserved throughout all kingdoms of life.

### The ribosome is a GEF for EF-G

On the basis of measurements of the affinities of GDP and GTP for free EF-G [12], it has been assumed that EF-G in the GTP-bound form binds to the pre-translocation (preT) ribosome, and that exchange of GDP for GTP occurs after release of EF-G•GDP from the ribosome [1,3]. In this work, however, we found the dissociation constant for the EF-G•GDP complex (K_GDP _= 9 μM) to be more than 60 times smaller than the dissociation constant for the EF-G•GTP complex (K_GTP _> 0.6 mM) (Figure 2a,b). Given that the GTP:GDP ratio in the living cell is only about 7:1 [13], a major fraction of EF-G is expected to be bound to GDP *in vivo *(Figure 2a,b). We also demonstrated that EF-G in complex with GDP can rapidly enter the preT ribosome, and that guanine-nucleotide exchange on EF-G occurs on, rather than off, the ribosome (Figure 2c and Materials and methods). This defines the preT ribosome as a previously unknown GEF for EF-G.

The crystal structures of guanine-nucleotide-free and GDP-bound EF-G are virtually identical [16], and it has been difficult to obtain the crystal structure of GTP-bound EF-G. Regarding solution structures, a study with small-angle X-ray scattering (SAXS) was performed by Czworkowski and Moore [4] on EF-G from *Thermus thermophilus*. They found the scattering data obtained from guanine-nucleotide-free, GDP-bound, GTP-bound and GDPCP-bound EF-G to be virtually identical and very close to the virtual scattering curve calculated from the crystal structure of GDP-bound *T. thermophilus *EF-G. They estimated the dissociation equilibrium constants for the binding of GDP, GTP and the non-cleavable GTP analog GDPCP as 0.92, 14.1 and 790 μM, respectively. Unfortunately, the GTP-binding experiments were performed without further purification of the GTP solution, meaning that it contained an unknown fraction of GDP. Accordingly, they may have significantly overestimated the affinity of EF-G for GTP, which appears likely in light of our new results (Figure 2a,b). Even though their SAXS experiments were performed in the presence of an energy-regeneration system, pumping GDP back to GTP, the fraction of GDP was significant (2–5%) [4]. It could therefore be that the scattering data reflected EF-G molecules primarily bound to GDP. Our data (Figure 2a,b) do not exclude the possibility that a small fraction of EF-G in the cell is GTP-bound. As shown in Additional data file 1 with the online version of this article, this does not necessarily mean that the conformation of EF-G has switched to its GTP-bound form, in line with the close similarity of a very recent crystal structure of an EF-G mutant in complex with GDPNP [17] or the crystal structure of EF-G bound to GDP [18,19]. Furthermore, the mechanism we propose with ribosome-dependent guanine-nucleotide exchange makes all EF-G molecules active in ribosome binding independent of their guanine-nucleotide content, and effectively prevents idling GTPase activity of EF-G [8].

### Guanine-nucleotide exchange requires the 'twisted' ribosome conformation

**Figure 8:**
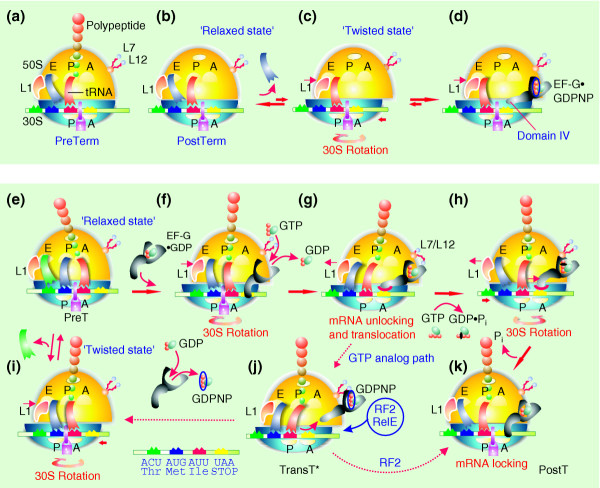
Interaction between the ribosome and EF-G; a proposal for the whole mechanism of translocation. **(a-c) **Release of peptide from the postTerm ribosome allows the intersubunit rotation. **(d) **EF-G•GDPNP stabilizes the twisted form of the ribosome as observed by cryo-EM [5]. **(e-h,k) **A model explaining the mechanism of translocation. **(i,j) **A GTP-analog pathway. The model is explained in detail in the text and symbols are as in Figure 1. L7/12 is a complex of ribosomal proteins thought to activate GTP hydrolysis on ribosomal GTPases. The mauve padlock illustrates states of the ribosome in which the mRNA is locked, and cannot move in relation to the small subunit. Domain IV of EF-G is suggested in the main text to play an important role in translocation.

Cryo-EM studies [5] have shown that the deacylated tRNA in the P site of the post-termination (postTerm) ribosome moves to the hybrid P/E site when the ribosome forms a high-affinity complex with EF-G•GDPNP (Figure 8d). During this movement, the 30S subunit is rotated (twisted) in relation to the 50S subunit. The affinity of EF-G•GDPNP for the post-translocation (postT) ribosome with a peptidyl-tRNA in the P site (Figure 8a,k) is weak [8]. A peptidyl-tRNA, in contrast to a deacylated tRNA, cannot adapt to the P/E hybrid site, and it was therefore concluded that high-affinity binding of EF-G•GDPNP (or EF-G•GTP) to the ribosome requires that the ribosome is in the twisted conformation [5,8]. This suggests that guanine-nucleotide exchange on EF-G and the concomitant conformational switch from its GDP- to its GTP-bound form during translocation must take place when the ribosome is in the twisted conformation with hybrid sites for its tRNAs. If so, this would be analogous to the mechanism by which class-1 release factors are recycled by RF3. In that case, it was found that the GEF for RF3 is the postTerm ribosome (Figure 8b) with a deacylated tRNA in the P site and a class-I peptide-release factor in the A site [9,10]. Before discussing the experimental evidence supporting this hypothesis, we will discuss what appears to be conflicting experimental evidence regarding the conformation of the preT ribosome.

Moazed and Noller [14] found that, after peptide-bond formation, a peptidyl-tRNA originally bound in the A site moves to the hybrid A/P site (Figure 8i). It was further suggested that the 30S ribosome must rotate relative to the 50S subunit to preserve the structure of the tRNA [20]. At the same time, reconstruction of the ribosomal preT complex using cryo-EM shows a relaxed state of the ribosome with three tRNAs in 'classical' A, P and E sites [5]; (Figure 8e). This apparent contradiction between two different sets of experimental results can, we suggest, be explained by different experimental conditions. The twisted preT state [14] was observed in the absence of free deacylated tRNAs (D. Moazed, personal communication), while the relaxed preT state was seen in their presence [5,8]. Because filling of the E site is incompatible with the twisted state (Figure 8i), the presence of deacylated tRNA in excess can drive the ribosome from its twisted [14] to its relaxed [5] preT state (Figure 8e).

In the present work, we demonstrate that deacylated tRNA binds to the E site of preT as well as postT ribosomes with low codon-anticodon specificity, and with affinities favored by low temperature (Figures 5a,b and 7a,b,e). These data can explain the presence of E-site-bound tRNA in the cryo-EM reconstructions of the relaxed preT (Figure 8e; [5]) and postTerm (Figure 8b; [21]) ribosome. Recent results [22] indicate that the tRNAs on the preT ribosome fluctuate between 'classical' A-P (Figure 8e) and hybrid A/P-P/E (Figure 8i) sites in an equilibrium that may depend on the occupancy of the E site, and therefore on the concentration of free deacylated tRNA.

We have now found that addition of EF-G•GDP to preT ribosomes reduces the affinities of deacylated tRNAs to the E site (Table 1). This supports the hypothesis that EF-G•GDP can drive a preT ribosome from its relaxed [5] to its twisted conformation [5,20] (Figure 8e,f), as this would reduce the apparent affinity of deacylated tRNAs for the E site. From this we propose that when EF-G•GDP encounters a relaxed preT ribosome, it induces a twisted ribosome conformation in which the exchange of GDP to GTP on EF-G takes place.

### EF-G in the GTP-bound form drives the ribosome into a transition state

When GDP is exchanged for GDPNP on EF-G in the preT ribosome, EF-G changes conformation and the ribosome moves from the preT state to the transition state transT* (Figure 8j). The transT* structure has not been observed directly, but some of its characteristics can be guessed from the cryo-EM reconstruction of the complex between EF-G•GDPNP and the postTerm ribosome (Figure 8d; [5]). Here, domain IV, located on the tip of EF-G and mimicking the anticodon end of tRNA [23], is positioned in the A site of the 30S subunit. The shape of EF-G•GDPNP is reminiscent of previous observations of the EF-Tu•GDP•aminoacyl-tRNA ternary complex, stalled in the A/T site of the postT ribosome with the antibiotic kirromycin [24]. In the A/T site, the anticodon end of the aminoacyl-tRNA is bound to the A site of the small ribosomal subunit, while its CCA end is bound to EF-Tu. We propose that in order to adopt a similar conformation on the preT ribosome, EF-G must displace the peptidyl-tRNA from the A site of the 30S subunit (Figure 8g). The transT* state is formed, we suggest, by translocation of the tRNA_2_-mRNA complex in relation to the 30S subunit. This would allow ribosomal RNA helix h44 and ribosomal protein S12 to interact with domain IV of EF-G [5], which could prevent slippage of the tRNAs back to their preT positions. The fundamental role that we propose for domain IV of EF-G is supported by previous observations that truncation of, or single point mutations in, domain IV inhibit translocation [25]. Furthermore, the finding that the affinity of EF-G•GDPNP to the transT* ribosome (Figures 5c,8j) is much lower than for the postTerm ribosome [8] can be explained by the proposed competition for binding to the A site between domain IV of EF-G•GDPNP and the anticodon part of peptidyl-tRNA in the former case (Figure 8j) but not in latter case (Figure 8d).

We have shown previously that peptidyl-tRNA, situated in the A site of the preT ribosome, becomes puromycin-reactive in the ribosomal transT* state, but that the rate of this reaction is much slower than for peptidyl-tRNA in the postT state (Figure 6b; [8]). The class-1 release factor RF1 or RF2, which normally binds to the stop codon of the preTerm state, here appearing in an identical conformation to the postT state (Figure 8k), also induces slow peptide release from the peptidyl-tRNA in the transT* ribosome (Figure 6c). This means that the stop codon, which in the preT ribosome is downstream from and adjacent to the A-site codon (Figure 8e), must be at least intermittently present in the A site of the transT* ribosome (Figure 8j). At the same time, dissociation of the peptide from the transT* ribosome in the presence of RF2 is considerably slower than peptidyl transfer to puromycin (Figure 6b,c), and much slower than RF2-dependent peptide release from the postT (Figure 8k) or preTerm (Figure 8a) ribosome [9,10]. These results suggest that EF-G•GDPNP blocks access of the release factor to the A site (Figure 3c). It is only when EF-G•GDPNP dissociates from the ribosome that RF2 can bind to the A site and induce peptide release (Figure 6c; [8]).

Further evidence that the mRNA moves one codon in relation to the 30S subunit when the ribosome switches from preT to transT* state comes from experiments with RelE. These reveal a cut in the stop codon of transT* ribosomes, implying that it must have moved at least intermittently into the A site (Figures 3b,6a). The mRNA cleavage is slow, suggesting that EF-G•GDPNP must dissociate before RelE can enter the ribosome and cut the mRNA (Figure 8j). After RelE cleavage of the mRNA, the ribosome remains in the transT* state (Figure 5d).

We have also demonstrated here that the deacylated tRNA, which was originally in the P site (Figure 8e), remains in contact with the mRNA in the transT* state and thus can be exchanged only with a deacylated tRNA of the same type (Figure 5a,b). This further indicates that the tRNAs in the transT* state have not been completely accommodated in the postT state where a deacylated tRNA bound to the E site would lack codon specificity (Figure 7a,b and Table 1).

### Ribosome movement from the transition state to the post-termination state

To understand how the ribosome moves from the transT* to the postT state, one must understand the role of GTP hydrolysis in the translocation process.

A striking finding of the work described here is that removal of EF-G•GDPNP from the transT* ribosome by addition of excess GDP does not lead to complete translocation, as would follow from the 'classical' model [4,6], but instead brings the ribosome back to the preT state (Figure 6). This means that, after dissociation of EF-G•GDPNP, the transT* structure is spontaneously pulled back to the preT, rather than the postT, structure of the ribosome (Figure 8e,i,j). The driving force for this movement is provided by the affinity of the anticodon end of the peptidyl-tRNA for the decoding center of the 16S rRNA, which is missing in the transT* and present in the preT state. Subsequent return to the transT* state by the action of EF-G is prevented by the presence of GDP (Figure 8i,j). When, in contrast, GTP is added to a transT* ribosome that is stabilized by EF-G•GDPNP, translocation is completed (Figure 8g,h,j).

Remarkably, there is another pathway to complete translocation. That is, when RF2 is added to a ribosome in the transT* state with EF-G•GDPNP which has a UAA stop codon in the A site of its postT state, the ribosome eventually ends up in the postTerm state with RF2 in the A site and deacylated tRNA in the P site (Figure 8k). This means that the driving force, generated by the strong affinity of RF2 for the relaxed postTerm ribosome [10], is sufficient to overcome the counteracting force provided by the affinity of the anticodon end of the peptidyl-tRNA for the decoding center in the preT ribosome. When GTP is hydrolyzed on EF-G in the transT* ribosome (Figure 8g,h,k), the elongation factor must adopt a conformation that can do the same trick as RF2.

This conformation of EF-G in Figure 8h cannot be the structure of EF-G•GDPNP [5] because the latter has very low affinity for the postT ribosome [8]. Instead, we suggest that GTP hydrolysis on EF-G brings EF-G•GDPNP from its GTP-bound form, with high affinity for the twisted transT* ribosome and low affinity for the relaxed postT ribosome [8], to an EF-G•GDP•P_i _form that has high affinity for the relaxed postT ribosome. During this conformational change, the 30S subunit is pulled by domain IV of EF-G into the relaxed conformation with docking of the tRNA_2_-mRNA complex in the postT state (Figure 8k). In this step, the deacylated tRNA in the hybrid P/E site of the transT* ribosome loses contact with the mRNA and moves into the E/E site (Figures 5a,b and 7; [15]). It is possible that the GDP•P_i _form of EF-G is similar to the EF-G•GDP form found in postT ribosomes in the presence of fusidic acid [5]. When inorganic phosphate is released from EF-G in the absence of fusidic acid, EF-G adopts the free GDP-bound conformation with low affinity to the postT state, and rapidly dissociates from the ribosome. When, in contrast, fusidic acid is present, EF-G remains in the EF-G•GDP•P_i _form, stably bound to the postT ribosome.

### Comparison with previous models

Our proposal that EF-G in the GTP form drives the ribosome into a transition state (transT*), with properties distinct from those of the preT and postT ribosome, contrasts with the 'motor' mechanism proposed by Rodnina *et al. *[7], in which the action of EF-G comes after GTP hydrolysis. We have also identified the GTP-bound conformation of EF-G in the transition state with the cryo-EM reconstruction of EF-G•GDPNP on the postTerm ribosome [5].

Our proposal differs further from the classical model [4,6] as well as from the model proposed by Rodnina *et al. *[7] in that movement of the ribosome to the postT state requires both GTP and GTP hydrolysis, and cannot occur with EF-G in the presence of a GTP analog or GDP. The present finding that complete translocation to the postT ribosome requires GTP hydrolysis is compatible with the observation that GTP hydrolysis precedes translocation [7]. We suggest further that the GDP-dependent translocation observed by Rodnina *et al. *[7] originates in a GTP-contaminated GDP solution, which would result in exactly the slow, monophasic translocation they observe (see Materials and methods for further details).

Our model proposes roles for the EF-G•GDP•P_i _structure and release of inorganic phosphate that are distinct from previous suggestions [7]. That is, we suggest that GTP hydrolysis on EF-G in the ribosomal transT* state results in a conformation of EF-G•GDP•P_i _that catalyzes ribosome movement from the transition state to the postT state. Then, dissociation of EF-G from the ribosome requires release of inorganic phosphate (P_i_), which favors formation of the GDP-bound structure of EF-G [16] with low affinity for the ribosome [8]. If it is assumed that the conformation of EF-G in the EF-G•GDP•P_i _complex is the same as the conformation of EF-G in the EF-G•GDP•fusidic acid complex [5], then the mechanistic action of fusidic acid could be rationalized as a freezing of EF-G in the conformation that catalyzes the second major step of translocation, bringing the ribosome from the transT* state to the postT state. In contrast, Rodnina and collaborators [26] associate release of inorganic phosphate with a 'relocking' conformational change of the ribosome, which leads to the postT state.

## Conclusion

The mechanism of translocation in eubacteria that we have suggested differs radically from all previous models [4,6,7,26,27] in that we propose the following: first, that EF-G enters the preT ribosome in the GDP-favoring form [16]; second, that EF-G•GDP drives the preT ribosome from its relaxed state with full binding sites for three tRNAs [5] to a twisted conformation with hybrid sites for two tRNAs [5]; and third, that exchange of GDP for GTP and an accompanying switch of the EF-G conformation from its GDP-bound to its GTP-bound structure occur on, rather than off, the ribosome.

Our results suggest that the ribosome plays a previously unidentified dual role of both guanine-nucleotide exchange factor and GTPase-activating protein for at least two translation factors in eubacteria, EF-G and RF3. This may be rationalized by the requirement that ribosomal GTPase activity be strictly controlled [8] and that each of the translation factors must selectively target a particular state of the ribosome.

## Materials and methods

### *E. coli *components for protein synthesis *in vitro*

Ribosomes of high activity, polymix buffer, initiation factors IF1, IF2 and IF3, translation factors EF-Tu, EF-Ts, EF-G and RF2, tRNA bulk, tRNA^Phe^, tRNA^Ile ^and overexpressed fMet-tRNA^fMet^, PheRS, ThrRS and IleRS were prepared as described [9,28]. The tRNAs were further purified by HPLC according to Cayama *et al. *[29] to aminoacylation activities greater than 80%. [^3^H]-GDPNP (Amersham Bioscience, Uppsala, Sweden) and other nucleotides (Sigma, Stockholm, Sweden) were further purified on a MonoQ column (Amersham Bioscience) as described ([9]; Figure 4a). They were bound to the MonoQ in Buffer A (20 mM Tris-HCl) and eluted with NaCl in buffer B (20 mM Tris-HCl and 1 M NaCl). The Met-Ile-encoding mRNA used to make translocation complexes had the sequence gggcccuuguuaacaau**uaaggaggu**auxxx AUG AUU **UAA **uugcag(a)_21_. It contained a strong ribosome-binding site, an open reading frame encoding a Met-Ile dipeptide (capital letters), a stop codon **UAA **and a poly(A) tail for purification on a poly(dT) column; xxx was either a Thr (acu) or a Phe (uuu) codon. The mRNA encoding the Met-Phe-Thr-Ile tetrapeptide had the open reading frame AUG UUU ACG AUU. The mRNAs were synthesized by T7 RNA polymerase transcription of DNA oligonucleotides. [^33^P]-labeling of tRNA^fMet ^and mRNAs followed standard protocols (Amersham Bioscience).

### Ribosomal complexes

Init and preT complexes were prepared according to Zavialov and Ehrenberg [8]. PostT and preTerm complexes were obtained according to Zavialov *et al. *[9]. In some experiments either [^33^P]-labeled mRNA [11] or [^33^P]-tRNA^fMet ^was used for complex preparation. The fraction of peptidyl-tRNA in the complexes was more than 80% (for Init, postT and preTerm complexes) or 70% (for preT).

### Binding of GDP, GTP and GDPNP to EF-G

For the experiment shown in Figure 2a, 5 μM EF-G was incubated for 2 min at 37°C with [^3^H]-GDP (3–18 μM) in 50 μl. The tubes with EF-G and GDP were then put on ice. After 15 min, 45 μl of each sample was nitrocellulose-filtered and washed with 500 μl ice-cold polymix buffer. All operations were done in a cold room (4°C). The amount of EF-G• [^3^H]-GDP retained on the filter was quantified by scintillation counting [9]. For the experiment shown in Figure 2b, 5 μM EF-G was incubated with 45 μM [^3^H]-GDP and unlabeled GTP (0–2.5 mM) or GDP (0–1.2 mM). Other conditions were the same as in Figure 2a. For the experiment shown in Figure 2d, 2 μM EF-G, 92 nM 70S ribosomes with 1 μM Met-Phe-Thr-Ile mRNA or 92 nM preTerm complexes with 0.4 mM puromycin were incubated with 1 μM [^3^H]-GDPNP in the presence of cold GDP (0–2 mM) for 7 min at 37°C in 50 μl. Then, 45 μl of each sample was nitrocellulose-filtered. For the experiment shown in Figure 2e, 2 μM EF-G, 92 nM 70S ribosomes with 1 μM Met-Phe-Thr-Ile mRNA or preTerm complexes with 0.4 mM puromycin were pre-incubated with 1 μM [^3^H]-GDPNP for 4 min at 37°C and then 2 mM GDP (final concentration) was added. After GDP addition 45 μl aliquots were nitrocellulose-filtered. For Figure 2f, EF-G was either incubated for 4 min at 37°C with preTerm complexes, puromycin and [^3^H]-GDPNP and then buffer or RF2 was added, or alternatively, [^3^H]-GDPNP was added to EF-G pre-incubated with preTerm complexes, puromycin and RF2. Then, 50 μl aliquots were nitrocellulose-filtered to determine the amount of EF-G• [^3^H]-GDPNP bound to the postTerm complex. The final concentrations of components in the mixtures were: 92 nM preTerm, 0.4 mM puromycin, 2 μM EF-G, 1 μM RF2 and 1 μM [^3^H]-GDPNP.

### Toxin inducible cleavage of mRNA (TICOM)

For the experiment shown in Figure 3a, 0.12 μM RelE was incubated for 1 min at 37°C with 0.2 μM initiation complex and 0.12 μM preT complex or with 0.24 μM preT complex, 2 μM EF-G and GTP in 50 μl. Then, 10 μl of 'kill' mix (1.25% SDS and 0.25 M EDTA pH 8.1) was added to stop the reaction. The samples were phenol-extracted, precipitated with ethanol and the RNA fragments were run in a sequencing gel (10% polyacrylamide, 8 M urea; [11]). The amount of mRNA cleaved by RelE, normalized to the total intensity of RNA bands in the lane, was determined using the "ImageQuant5.0" program (Amersham Bioscience). The background hydrolysis of mRNA was used as a nucleotide ladder. For Figure 3b, either RelE was added to preT ribosomes incubated with EF-G and one of the nucleotides at different times and then the resulting mix was incubated 5 min more before quenching, or RelE was always present in the mix. The final concentrations of components in the mix (50 μl) were: 0.1 μM RelE, 2 μM EF-G, 0.15 μM preT and 1 mM of each nucleotide. In all experiments cleavage in the UAA codon in the presence of EF-G and GTP was set at 100%. For Figure 3c, 120 nM RelE was incubated with 0.3 μM postT or preT complex, 2 μM EF-G and 0.6 mM GDPNP at 37°C, and 50 μl aliquots were removed and analyzed at different time points (0–4 min). At 4 min, 100 mM GTP was added to obtain 1 mM final concentration and the incubation continued for 1 min before quenching.

For Figure 4c, 80 nM RelE was incubated for 3 min at 37°C in 50 μl with 2 μM EF-G, 0.15 μM preT and nucleotides as indicated in the figure.

For Figure 6a, EF-G, GDPNP and preT were incubated at 37°C for 3 min. Then, the mix was divided into two tubes with GDP in one and buffer in the other. The mixes were then incubated with RelE at 37°C and 50 μl aliquots were analyzed at 15 and 28 min of incubation. After 29 min GTP was added and the incubation continued for 1 min before quenching. The final concentrations of the components were 2 μM EF-G, 40 μM GDPNP, 0.4 μM preT, 166 nM RelE, 2 mM GDP and 1 mM GTP.

### Release of [^33^P]-tRNA^fMet ^from the pre-translocation complex

For the experiment shown in Figure 5a, EF-G and a nucleotide were pre-incubated with preT complex containing [^33^P]-tRNA^fMet ^in the P site for 3 min at 37°C. Then, cold tRNA^fMet ^or tRNA^Phe ^was added and 45 μl aliquots were nitrocellulose-filtered at different times to determine the fraction of [^33^P]-tRNA^fMet ^on the ribosome. The concentrations of components in the mix were: 70 nM preT, 2 μM EF-G, 1 μM cold tRNA^fMet ^or tRNA^Phe ^and 1 mM nucleotide. For Figure 5b, EF-G, a nucleotide, preT and 0–2 μM tRNA^fMet ^or tRNA^Phe ^were pre-incubated for 9 min at 37°C. Then, 45 μl aliquots were nitrocellulose-filtered and washed with 1 ml polymix buffer. For Figure 5c, 2 μM EF-G was incubated for 7 min at 37°C with 88 nM preT, 2 μM tRNA^fMet ^ and GDPNP (0–240 μM). Then, 45 μl aliquots were nitrocellulose-filtered. For Figure 5d, preT complexes with [^33^P]-tRNA^fMet ^in the P site were pre-incubated with EF-G and GDPNP, with or without RelE, for 3 min at 37°C. Then, cold tRNA^fMet ^or tRNA^Phe ^was added and 45 μl aliquots were nitrocellulose-filtered. After 27.5 min incubation GTP was added to the mix, which then contained 78 nM preT, 2 μM EF-G, 0.4 mM GDPNP, 2 μM tRNA^fMet ^ or tRNA^Phe^, 1 mM GTP and 80 nM RelE.

For Figure 6d, EF-G was pre-incubated for 3 min at 37°C in polymix buffer with or without preT and GDPNP. Then, GDP or polymix buffer was added. After 1 min incubation, tRNA^fMet ^was added and then 45 μl aliquots were nitrocellulose-filtered after different times. The final mix contained 88 nM preT,2 μM EF-G, 100 μM GDPNP, 1 μM tRNA^fMet ^and 2 mM GDP.

### Release of [^3^H]-fMet-[^14^C]-Ile peptide by RF2 or puromycin

For the experiment shown in Figure 4b, mix A, containing 1 μM EF-G, 23 nM preT complex and 0.4 μM RF2, was pre-incubated for 3 min at 37°C. Then mix B, containing nucleotides, was added to mix A, and 45 μl aliquots were removed at different time points for determination of the amount of released peptide according to Zavialov and Ehrenberg [8]. The resulting mix contained 1 μM EF-G, 23 nM preT, 0.4 μM RF2 and nucleotides as indicated in the figure.

For Figure 2c, mix A containing EF-G, GTP, RF2, and varying GDP concentrations was pre-incubated for 3 min at 37°C. Then, mix B containing preT complex was added and the amount of released peptide in 45 μl aliquots was determined at different time points. The resulting mix contained 10 nM EF-G, 23 nM preT, 0.4 μM RF2, 0.5 mM GTP and 0–0.8 mM GDP.

For Figure 6b and c, EF-G was pre-incubated for 3 min at 37°C with preT complex and GDPNP or polymix buffer. Then, GDP or buffer was added, the incubation was continued for 1 min, and then puromycin (Figure 6b) or RF2 (Figure 6c) was added and the amount of released peptide in 45 μl aliquots was determined at different time points. The final mix contained 2 μM EF-G, 46 nM preT, 0.5 μM RF2 or 0.4 mM puromycin and nucleotides as indicated on the figure.

### Binding of tRNAs to the E site

Typically, a ribosomal complex (10–30 nM) was incubated with [^33^P]-tRNA^fMet ^dilutions for 5 min at 37°C and then 45 μl of the mix was nitrocellulose-filtered in order to estimate the amount of ribosome-bound [^33^P]-tRNA^fMet^. To observe the effect of A-site cleavage of mRNA, the incubation was carried out with 100 nM RelE. To measure the binding of [^33^P]-tRNA^fMet ^to the E site of postT ribosomes at 0°C, tubes with the mix were incubated on ice for 20 min before the filtration. To measure the binding (exchange) of [^33^P]-tRNA^fMet ^to preT ribosomes in the presence of EF-G, 3 μM EF-G and 1 mM GDPNP, GTP or GDP were added to the reaction mix. To determine the dissociation constant (K_I_) for the binding of tRNA^Phe ^to the E site from the inhibition curves in Figure 7b, a ribosomal complex was incubated with 0.8 μM [^33^P]-tRNA^fMet ^and increasing concentrations of unlabeled tRNA^Phe ^K_I _values were obtained from K_I _= I50/(1 + [tRNA ^fMet^]/Kd), where I_50 _is [tRNA^Phe^] at 50% inhibition of tRNA^fMet ^binding (dissociation constant K_d_).

### Translocation kinetics with mixtures of GDP and GTP

#### Experimental analysis of EF-G•GDP binding to the preT ribosome

In the experiment shown in Figure 2c, EF-G is recycling from ribosome to ribosome, catalyzing translocation, and the ratio between GDP and GTP is varied. Under this condition of ribosomes in excess, one can formally describe EF-G as an enzyme and the ribosome as its substrate and apply text-book enzyme kinetics [30].

If only EF-G in the GTP-bound form can bind the ribosome, and if we only consider EF-G bound to either GTP or GDP, neglecting nucleotide-free EF-G, then the steady state flow of translocation [30] can be written as



Here, [*EF-G*_0_] and [70*S*_0_] are total concentrations of EF-G and 70S ribosomes, respectively; *k*_*cat *_is the maximal rate of the EF-G cycle at saturating ribosome concentration; *K*_*m *_is the *K*_*m *_value of this cycle in the absence of GDP; *K*^*GTP *^and *K*^*GDP *^are dissociation constants for the binding of GTP or GDP to free EF-G, respectively. As written in the main text, this experiment was performed under conditions where the flow *j *is a linear function of the total ribosome concentration, meaning that [70*S*_0_] <<*K*_*m*_, so that *j *can be written



We know that the ratio between *K*_*GTP *_and *K*_*GDP *_is larger than 60 (Figure 2b), from which it follows that inhibition of the recycling rate should be larger than 30 at a [GDP]: [GTP] ratio of 0.5. But given that the observed inhibition at this nucleotide ratio is only a factor of two, it follows that EF-G in the GDP-bound form must be able to enter the ribosome. There is, in fact, no other way to explain the combination of results in Figure 2a–c.

If, in contrast, EF-G can enter the ribosome in the GDP-bound form, and guanine-nucleotide exchange takes place on rather than off the ribosome, the experimental results in Figure 2a–c are readily explained. One such scheme is presented in the next subsection (equation 3).

#### Ribosome kinetics with guanine-nucleotide exchange on the ribosome

A simple kinetic scheme for translocation with guanine-nucleotide exchange on the ribosome is



EF-G enters the preT ribosome in complex with GDP with an association rate constant *k*_*a*_. In the next step, EF-G•GDP may dissociate from the ribosome (rate constant *k*_*d*_) or, alternatively, GDP dissociates from EF-G (rate constant ). Ribosome-bound guanine-nucleotide-free EF-G can either bind GDP (compounded rate constant  [*GDP*]) or GTP (compounded rate constant  [*GTP*]). GTP-bound EF-G then promotes translocation (rate constant *k*^*T*^). To simplify, we have neglected dissociation of guanine-nucleotide free EF-G from the ribosome. The Michaelis-Menten parameter *k*_*cat*_/*K*_*m *_for this scheme is given by



Interpreted in terms of this scheme, the two-fold reduction in the rate of cycling of EF-G at 0.5 mM GTP and 0.25 mM GDP shown in Figure 2c, simply means that



The *k*_*cat *_value for the scheme, corresponding to the rate of translocation at saturating concentration of EF-G (single-round kinetics) is given by



The first term on the right side of this equation is the average time for GDP to dissociate from EF-G (1/*k*_*d*_^*GDP *^) multiplied by the average number of dissociations of GDP per successful translocation.

Rodnina *et al. *[7] found monophasic kinetics when they measured translocation in the presence of an unpurified solution of GDP that is likely to contain a trace amount of GTP. They monitored translocation with EF-G in excess over the ribosome, in which case our model predicts monophasic kinetics with a much slower rate than when GTP is in large excess over GDP. To see this, we first note that when the [GDP]: [GTP] ratio is large, the *k*_*cat *_value for translocation simplifies to



To show that in this limiting case translocation occurs with a single first-order rate constant equal to *k*_*cat*_, we denote the concentration of the preT ribosome in complex with EF-G•GDP by *c*_1_, in complex with guanine-nucleotide-free EF-G by *c*_2_, and set *c *= *c*_1 _+ *c*_2_. At saturating free concentration of EF-G•GDP we have *c *= *c*_1 _+ *c*_2 _≈ *c*_1 _and



Furthermore,



This shows that single-round translocation in a system with a large concentration of GDP and with a small contamination of GTP is expected to be approximated by a single exponential



as observed in the experiment carried out by Rodnina *et al. *[7].

## Additional data files

The following is provided as an additional data file with the online version of this article. Additional data file 1, showing that EF-G in solution can change from a GDP to a GTP conformation.

## Supplementary Material

Additional File 1 An additional data file showing that EF-G in solution can change from a GDP to a GTP conformation Click here for file

## Figures and Tables

**Table 1:** Dissociation constants for the binding of tRNA^fMet ^and tRNA^Phe ^to different ribosomal complexes

Set	State	Peptide	Codon in the E site	tRNA	Dissociation constant K_D _(nM)	Additional factors; temperature
1	PreT	fMI	Thr (ACG)	fMet	144 ± 7	37°C
					179 ± 14	EF-G + GTP; 37°C
					770 ± 80	EF-G + GDP; 37°C
					40 ± 2	EF-G + GDPNP; 37°C
2	PostT	fMI	Met (AUG)	fMet	153 ± 9	37°C
				Phe	250 ± 30	
		fM	Phe (UUU)	fMet	295 ± 26	
				Phe	84 ± 11	
		fMFTI	Thr (ACG)	fMet	162 ± 5	
				Phe	134 ± 20	
3	PostT	fMI	Met (AUG)	fMet	155 ± 10	+ RelE (A-site cut); 37°C
4	PostT	fMI	Met (AUG)	fMet	24.8 ± 1.2	0°C

Different conditions were used for measuring the dissociation constants for the different combinations of tRNA and ribosomal complexes as in Figure 7, and are shown in the last column.
