# Supplementary material for: Guanine-nucleotide exchange on ribosome-bound elongation factor G initiates the translocation of tRNAs
Source: J Biol. 2005 Jun 27;4(2):9. doi: 10.1186/jbiol24 (PMC1175996; doi:10.1186/jbiol24)
Supplement: Additional File 1 — An additional data file showing that EF-G in solution can change from a GDP to a GTP conformation [file jbiol24-s1.pdf]

Additional data file 1 (added in proof)

Can free EF-G in solution change from a GDP to a GTP conformation?

The crystal structures of guanine-nucleotide-free- and GDP-bound EF-G are very similar [18,19], and small-angle X-ray scattering (SAXS) data suggest that the solution structures of EF-G and EF-G•GDP are virtually identical and very similar to their crystal structures [4]. Interestingly, the same SAXS data also suggest that the solution structure of GTP-bound EF-G might be indistinguishable from the guanine-nucleotide-free- and GDP-bound forms of the factor in solution as well as in crystal [4]. Very recently the notion that the solution structures of EF-G•GTP and EF-G•GDP are themselves similar yet are very different from the GDPNP-bound form of the factor on the post-termination ribosome [5] received support from a novel crystal structure of the GTP-bound form of a mutant of EF-G from *Thermus thermophilus* [17]. Could it then be that the GDP- and GTP-bound forms of the free factor are similar, while they are very different on the ribosome?

To examine the conditions for such a scenario, one may introduce a GDP-favoring (R) and a GTP-favoring (T) conformation of EF-G:

$$\begin{array}{ccc} RGDP & \underset{}{\overset{K_0/\ell^{GDP}}{\rightleftarrows}} & TGDP \\ K_R^{GDP}\downarrow\uparrow & & K_T^{GDP}\downarrow\uparrow \\ R & \overset{K_0}{\rightleftarrows} & T \\ K_R^{GTP}\downarrow\uparrow & & K_T^{GTP}\downarrow\uparrow \\ RGTP & \overset{K_0\cdot\ell^{GTP}}{\rightleftarrows} & TGTP \end{array} \qquad (1)$$

R and T are guanine-nucleotide-free R and T forms, respectively. RGDP is the R form in complex with GDP, TGDP is the T form in complex with GDP, RGTP is the R form in complex with GTP, and TGTP is the T form in complex with GTP. The parameters ℓ^{GDP} and ℓ^{GTP} are defined from the dissociation constants in Scheme 1 as

$$\ell^{GTP} = \frac{K_R^{GTP}}{K_T^{GTP}},\ \ell^{GDP} = \frac{K_T^{GDP}}{K_R^{GDP}} \tag{2}$$

We define effective dissociation constants for the binding of GTP or GDP to EF-G from the relations

$$\begin{aligned} \frac{([R]+[T])[GDP]}{K_{eff}^{GDP}} &= ([RGDP]+[TGDP]), \\ \frac{([R]+[T])[GTP]}{K_{eff}^{GTP}} &= ([RGTP]+[TGTP]) \end{aligned} \tag{3}$$

The effective dissociation constants K_{eff}^{GTP} and K_{eff}^{GDP} are given by the parameters of Scheme 1 through

$$\begin{aligned} K_{eff}^{GDP} &= \frac{K_R^{GDP}\ell^{GDP}(1+K_0)}{\ell^{GDP}+K_0}, \\ K_{eff}^{GTP} &= \frac{K_T^{GTP}\ell^{GTP}(1+K_0)}{1+\ell^{GTP}K_0} \end{aligned} \tag{4}$$

The probability, *P(T)*, that EF-G is in the T form depends on the GDP and GTP concentrations through

$$P(T) = \frac{K_0\left(1+\frac{[GTP]}{K_T^{GTP}}+\frac{[GDP]}{\ell^{GDP}K_R^{GDP}}\right)}{K_0\left(1+\frac{[GTP]}{K_T^{GTP}}+\frac{[GDP]}{\ell^{GDP}K_R^{GDP}}\right)+1+\frac{[GTP]}{\ell^{GTP}K_T^{GTP}}+\frac{[GDP]}{K_R^{GDP}}} \qquad (5)$$

The possibility that EF-G would switch in solution from its R to its T form by addition of pure GTP can be analyzed at zero GDP concentration in the limit of a large concentration of GTP in Equation (5):

$$P(T) = \frac{1}{1+\frac{1}{\ell^{GTP}K_0}} \qquad (6)$$

Accordingly, the condition for a switch is that $K_0 << 1$ and, yet that $\ell^{GTP}K_0 >> 1$. If, in contrast $\ell^{GTP}K_0 << 1$, then EF-G will remain in the R form in solution even at a very high concentration of pure GTP.

When EF-G binds to the pre-translocation ribosome, this could hypothetically lead to a large increase in K_0, so that $\ell^{GTP}K_0 << 1$ off and $\ell^{GTP}K_0 >> 1$ on the ribosome, allowing for a conformational switch on but not off the ribosome.

References

4. Czworkowski J, Moore PB: **The conformational properties of elongation factor G and the mechanism of translocation.** *Biochemistry* 1997, **36:**10327-10334.

5. Valle M, Zavialov A, Sengupta J, Rawat U, Ehrenberg M, Frank J. **Locking and unlocking of ribosomal motions.** *Cell* 2003, **114:**123-134.

17. Hansson S, Singh R, Gudkov AT, Liljas A, Logan DT: **Crystal structure of a mutant elongation factor G trapped with a GTP analog.** *FEBS Letters*. In press.

18. Ævarsson A, Brazhnikov E, Garber M, Zheltonosova J, Chirgadze Y, al-Karadaghi S, Svensson LA, Liljas A: **Three-dimensional structure of the ribosomal translocase: elongation factor G from *Thermus thermophilus*.** *EMBO J* 1994, **13:**3669-3677.

19. Czworkowski J, Wang J, Steitz TA, Moore PB: **The crystal structure of elongation factor G complexed with GDP, at 2.7 Å resolution.** *EMBO J* 1994, **13:**3661-3668.
